# Aquaglyceroporin-null trypanosomes display glycerol transport defects and respiratory-inhibitor sensitivity

**DOI:** 10.1371/journal.ppat.1006307

**Published:** 2017-03-30

**Authors:** Laura Jeacock, Nicola Baker, Natalie Wiedemar, Pascal Mäser, David Horn

**Affiliations:** 1 The Wellcome Trust Centre for Anti-Infectives Research, School of Life Sciences, University of Dundee, Dow Street, Dundee, United Kingdom; 2 Parasite Chemotherapy Unit, Swiss Tropical and Public Health Institute, Basel, Switzerland; 3 University of Basel, Basel, Switzerland; Oregon Health & Science University, UNITED STATES

## Abstract

Aquaglyceroporins (AQPs) transport water and glycerol and play important roles in drug-uptake in pathogenic trypanosomatids. For example, AQP2 in the human-infectious African trypanosome, *Trypanosoma brucei gambiense*, is responsible for melarsoprol and pentamidine-uptake, and melarsoprol treatment-failure has been found to be due to AQP2-defects in these parasites. To further probe the roles of these transporters, we assembled a *T*. *b*. *brucei* strain lacking all three *AQP*-genes. Triple-null *aqp1-2-3 T*. *b*. *brucei* displayed only a very moderate growth defect *in vitro*, established infections in mice and recovered effectively from hypotonic-shock. The *aqp1-2-3* trypanosomes did, however, display glycerol uptake and efflux defects. They failed to accumulate glycerol or to utilise glycerol as a carbon-source and displayed increased sensitivity to salicylhydroxamic acid (SHAM), octyl gallate or propyl gallate; these inhibitors of trypanosome alternative oxidase (TAO) can increase intracellular glycerol to toxic levels. Notably, disruption of *AQP2* alone generated cells with glycerol transport defects. Consistent with these findings, *AQP2*-defective, melarsoprol-resistant clinical isolates were sensitive to the TAO inhibitors, SHAM, propyl gallate and ascofuranone, relative to melarsoprol-sensitive reference strains. We conclude that African trypanosome AQPs are dispensable for viability and osmoregulation but they make important contributions to drug-uptake, glycerol-transport and respiratory-inhibitor sensitivity. We also discuss how the AQP-dependent inverse sensitivity to melarsoprol and respiratory inhibitors described here might be exploited.

## Author summary

Protein channels in cell membranes transport specific molecules in and out of cells, and can also facilitate drug-uptake. One such protein, known as an aquaglyceroporin (AQP), allows parasitic African trypanosomes, the cause of lethal diseases in humans and livestock, to accumulate an arsenic-based drug known as melarsoprol. Unfortunately, parasites with a mutated AQP have resisted this drug and have spread, leading to treatment-failure in >50% of patients in some areas. The functions of this particular AQP, and two other similar AQPs normally expressed by these parasites, remain to be fully characterised in trypanosomes. We therefore generated and characterised parasites lacking all three AQPs. The cells grow well and, to our surprise, continue to effectively allow water to flow in and out of the cell. Glycerol uptake and efflux are both perturbed, however. As a consequence, drugs that cause these parasites to produce toxic quantities of glycerol are more effective against parasites lacking the AQPs. Indeed, even the melarsoprol-resistant, patient-derived parasites described above are more sensitive to these drugs. Our findings not only reveal the relative contributions of the AQPs to glycerol transport, they also point to therapies that could be more effective in the many patients infected by melarsoprol-resistant parasites.

## Introduction

African trypanosomes are parasitic protozoa and the causative agents of human and animal African trypanosomiasis (HAT and AAT, respectively). These parasites are typically transmitted by tsetse-flies, which are restricted to sub-Saharan Africa. HAT is typically fatal without treatment, classified as a ‘neglected tropical disease’, and caused primarily by *T*. *brucei gambiense* (Western-Africa) but also by *T*. *brucei rhodesiense* (Eastern Africa). AAT is typically caused by *T*. *vivax*, *T*. *congolense* or *T*. *b*. *brucei*, important veterinary and livestock pathogens; *T*. *b*. *brucei* is a less-prevalent veterinary parasite and the favoured experimental sub-species. Vaccine development is challenging and therapies suffer problems with toxicity, resistance, cost, limited efficacy and difficulties with administration [1]. In addition, in the case of HAT, diagnostic tools must define the stage of the disease if the appropriate therapy is to be selected [1]. For treatment of the second stage for example, when parasites have entered the central nervous system, the nifurtimox-eflornithine combination therapy is favoured [2]. The other option is melarsoprol, but this is toxic [1]. Unfortunately, eflornithine is ineffective against *T*. *b*. *rhodesiense* [3] so melarsoprol is currently the only option, despite its toxicity, against advanced disease caused by this parasite.

Melarsoprol treatment-failure, in >50% of patients in some areas, has been reported for both *T*. *b*. *rhodesiense* [4] and *T*. *b*. *gambiense* infections [5]. Melarsoprol-resistance can arise due to reduced accumulation of drug, following aquaglyceroporin 2 (AQP2) mutation [6]. Both a trypanosome P2 adenosine transporter [7,8] and AQP2, an aquaglyceroporin with an unusual arrangement of pore-lining residues comprising the ‘selectivity filter’ [9,10], contribute to melarsoprol-uptake; laboratory-engineered defects in these transporters render cells melarsoprol-resistant. These cells also display cross-resistance to pentamidine [6], a drug used to treat trypanosomiasis prior to central nervous system involvement. This may have little impact in the clinic, however, because pentamidine remains effective at the high doses administered [11]. In terms of melarsoprol-resistance and treatment-failure, clinical isolates from both the Democratic Republic of the Congo and South Sudan, dating back to the 1970s, display *AQP2*-defects [12,13], and a clinical isolate was re-sensitised to both melarsoprol and pentamidine by the addition of an intact *AQP2* gene [14]. A defect in a related *Leishmania* AQP has been linked to widespread antimonial-resistant *Leishmania* infections in India [15].

There are three AQPs encoded in the *T*. *b*. *brucei* genome. AQP1 has been reported to localise to the flagellar membrane in bloodstream-form cells [16], while plasma membrane localisation is indicated in insect-stage cells [17]. AQP3 displays a plasma membrane localisation in both bloodstream-form cells [9,16] and insect-stage cells [9]. AQP2, on the other hand, is largely restricted to the flagellar pocket membrane in bloodstream-form cells, and then becomes distributed more widely in the plasma membrane in insect-stage cells [9]. Heterologous expression of the *T*. *b*. *brucei* AQPs reveals their ability to transport water, mass: 18 Da; ammonia, mass: 17 Da [18]; boric acid, mass: 62 Da [19]; glycerol, mass: 92 Da [20] and some forms of trivalent arsenic, mass: 83–198 Da; and trivalent antimony, mass: 122–292 Da [21]. *AQP2* gene-knockout in *T*. *b*. *brucei* reveals that this AQP can also specifically mediate uptake of melarsoprol; mass: 398 Da, and pentamidine; mass: 340 Da [9,10]. These drugs have a substantially greater mass than other known AQP-substrates and recent evidence indicates that pentamidine, rather than being a permeant, binds to and inhibits AQP2, suggesting that uptake of this drug might require endocytosis [22].

To further probe AQP-function, we deleted all three *T*. *b*. *brucei AQP* genes from the *T*. *b*. *brucei* genome. We found that trypanosomes tolerate the loss of all three *AQPs*. The triple *aqp1-2-3* null-strains, surprisingly, tolerated hypotonic shock, but were defective in glycerol uptake, utilisation and efflux and, consequently, were sensitised to trypanosome alternative oxidase (TAO) inhibitors that increase the intracellular glycerol concentration to toxic levels. Notably, trypanosomes lacking only *AQP2* were also defective in glycerol utilisation and efflux and, as predicted by our *T*. *b*. *brucei* studies, clinical melarsoprol-resistant *T*. *b*. *gambiense* isolates were also more sensitive to respiratory inhibitors relative to melarsoprol sensitive reference strains.

## Results

### *T*. *b*. *brucei* tolerates the loss of all three *AQPs*

**Fig 1:**
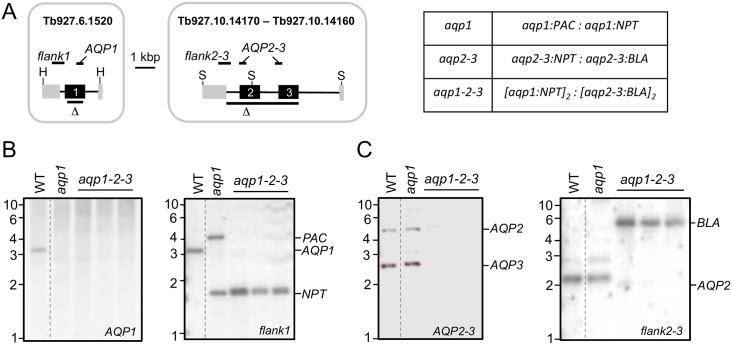
*T*. *b*. *brucei* tolerates the loss of all three *AQPs*. (A) The schematic maps indicate the *AQP1* and *AQP2-3* regions replaced by selectable markers as also indicated on the right. Δ indicates the regions deleted while the probes used for Southern blotting are shown above the maps. H, *Hpa*I; S, *Sac*II. (B) The Southern blots indicate deletion of the *AQP1* alleles in *aqp1* and three independent *aqp1-2-3* strains. Wild-type (WT) is shown for comparison. Genomic DNA was digested with *Hpa*I. (C) The Southern blots indicate deletion of the *AQP2-3* alleles in *aqp1-2-3* strains. WT is shown for comparison. Genomic DNA was digested with *Sac*II.

*T*. *b*. *brucei AQP1* (Tb927.6.1520) is on chromosome 6 and *AQP2* (Tb927.10.14170) and *AQP3* (Tb927.10.14160) are adjacent to each other on chromosome 10 (see Fig 1A). The *AQP2-AQP3* locus is dispensable for growth [23]. *AQP1* knockdown, using RNA interference was not associated with any substantial growth-defect [16], but knockout of *AQP1* has not, to our knowledge, been attempted. *T*. *b*. *brucei* is diploid so we sequentially replaced the *AQP1* alleles with selectable markers (*NPT* and *PAC*) to determine whether *AQP1* was dispensable (see Fig 1A). We readily obtained *aqp1*-null strains, as confirmed by Southern blotting (Fig 1B).

We next devised a strategy to assemble triple *aqp*-null strains in a background that would facilitate conditional expression of wild-type or mutant *AQPs* for complementation studies. In order to recycle the limited number of selectable-markers available, we used a multi-step strategy employing the meganuclease, I-*Sce*I (see Materials and methods). Briefly, we set up strains in the 2T1-background [24] in which meganuclease induction triggered the replacement of a chromosomal knockout-cassette, bearing an I-*Sce*I cleavage-site, with an allelic knockout-cassette lacking an I-*Sce*I cleavage-site. The cassette-integration and chromosomal allele-replacement process was carried out for the *AQP2-AQP3* locus and then repeated for the *AQP1* locus, such that the resulting strains bore a *BLA*-marker at both *aqp2-aqp3* null alleles and an *NPT*-marker at both *aqp1* null alleles (Fig 1A). Southern blotting confirmed the absence of *AQP1* (Fig 1B), *AQP2* and *AQP3* (Fig 1C) in the resulting *aqp1-2-3* null strains. Thus, *T*. *b*. *brucei* tolerates the loss of all three *AQPs*.

### The *T*. *b*. *brucei* AQPs have minimal impact on fitness or osmoregulation

**Fig 2:**
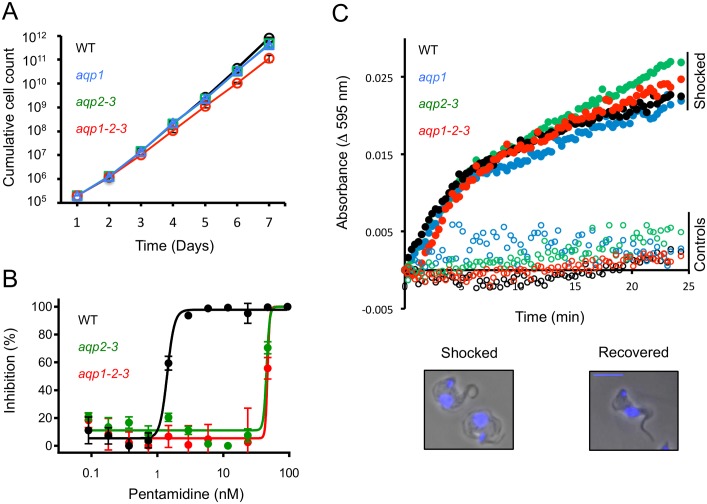
The *T*. *b*. *brucei* AQPs have minimal impact on fitness or osmoregulation. (A) Cumulative growth-curves for wild-type (WT), *aqp1*, *aqp2-3* and *aqp1-2-3* null-strains. (B) Dose-response curves for pentamidine. (C) Hypo-osmotic shock assay. Open symbols, Earle’s salt buffer; filled symbols, buffer diluted 50:50 with H_2_O. The recovery phase is shown. The phase-contrast images show two shocked and swollen cells (at left) and a recovered cell (at right). Scale-bar, 5 μm. DNA was counter-stained with DAPI (blue).

We assessed fitness in cell-culture for the new *aqp1* and *aqp1-2-3* strains and compared these to the wild-type and the previously described *aqp2-3* strains [9]. The growth-curves indicated a modest defect in the *aqp1-2-3* strains and no apparent defect in the *aqp1* or in the *aqp2-3* strains (Fig 2A). The *aqp1-2-3* strains were also able to establish infections *in vivo* in a mouse model; parasitaemia in all three mice was between 4 x 10^6^ and 4 x 10^7^ per ml of blood four days after inoculation. These *aqp1-2-3* strains also differentiated to the insect mid-gut stage *in vitro*; equivalent to wild-type after one week in insect-stage growth-medium. Thus, we observed only a modest fitness-defect in bloodstream-form cells in the absence of all three *AQPs* but not in the absence of either *AQP1* or *AQP2-AQP3*.

AQP2 specifically controls melarsoprol and pentamidine-uptake and has a particularly pronounced impact on pentamidine-sensitivity *in vitro* [9]. Dose-response assays confirmed the expected pentamidine-resistance in the *aqp2-3* strains and indicated no additional resistance in the *aqp1-2-3* strains (Fig 2B); EC_50_-values were increased by approximately 30-fold relative to wild-type in both cases. These results are consistent with the established specific role for AQP2 in pentamidine (and melarsoprol) uptake and cross-resistance [9,12,23].

AQPs can transport water or small solutes. To explore the contribution of the *T*. *b*. *brucei* AQPs to osmoregulation, we exposed cells to hypo-osmotic shock and monitored the response. Under these conditions, cells swell rapidly and then, more slowly (10–20 min), return to their original volume. We saw no, or only moderate, differences in the time taken to recover for *aqp1*, *aqp2-3 or aqp1-2-3* null-cells relative to wild-type trypanosomes (Fig 2C). We conclude that the *T*. *b*. *brucei* AQPs have minimal impact on fitness or regulatory volume-decrease after osmotic shock.

### Glycerol uptake and utilisation are perturbed in *aqp* null *T*. *b*. *brucei*

**Fig 3:**
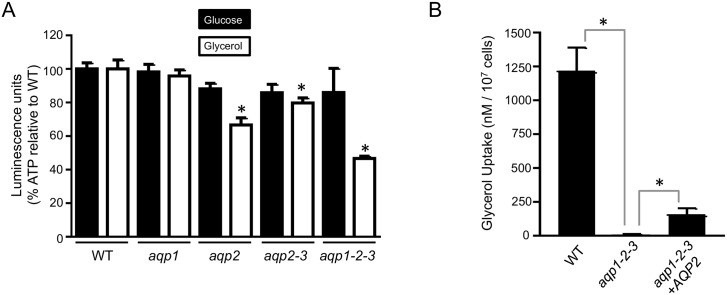
Glycerol uptake and utilisation is perturbed in *aqp*-null *T*. *b*. *brucei*. (A) ATP levels were assessed in the strains indicated after incubation in 5 mM glucose or glycerol. Readings were taken in triplicate and normalised to substrate only. * indicates significantly different (*P*<0.001) to wild-type (WT) using an ANOVA test in GraphPad Prism. Error bars, SD. (B) Radiolabelled glycerol uptake was assessed in the strains indicated. Readings were taken in quadruplicate. * indicates significant difference (*P*<0.05) using a Student’s *t*-test. Error bars, SD.

We next assessed the ability of the *aqp1-2-3* null *T*. *b*. *brucei* strains to use glycerol as a carbon-source, which is possible in bloodstream form trypanosomes under aerobic conditions [25]. In preliminary experiments, *aqp1-2-3* cells displayed sustained motility in 5 mM glucose and fructose but these cells were immotile within 15-minutes in 5 mM glycerol. To quantify the ATP-levels in cells incubated in 5 mM glucose or glycerol, we used a luminescence assay and this confirmed that *aqp1-2-3* cells were able to use glucose as a carbon-source but were unable to utilise glycerol (Fig 3A). Since ATP-levels were significantly depleted (*P*<0.001) relative to wild-type in *aqp1-2-3* cells incubated in glycerol, we exploited this assay to assess the impact of the various AQPs on glycerol utilisation; cells were harvested before they became immotile in this assay so as to record quantitative differences among strains. As expected, ATP-levels were not significantly diminished in any of the *aqp*-defective strains tested in glucose (Fig 3A). In glycerol though, ATP-levels were significantly depleted (*P*<0.001) in *aqp2*, *aqp2-3* and *aqp1-2-3* cells but not in *aqp1* cells (Fig 3A). These results suggest that, among the AQPs, AQP2 makes the greatest contribution to glycerol utilisation; this interpretation is supported by both effective utilisation of glycerol by *aqp1* null cells and no increase in the glycerol-utilisation defect in *aqp2-3* cells relative to *aqp2* cells.

Since glycerol utilisation does not directly reflect glycerol uptake, we next measured glycerol uptake; in wild-type, triple-null and *AQP2*-complemented cells. The *aqp1-2-3* cells revealed almost complete ablation of glycerol-uptake (Fig 3B), consistent with minimal diffusion of glycerol across the plasma membrane. *AQP2* provided complementation of this defect, albeit only partial (Fig 3B). Thus, AQP2 appears to make the greatest contribution to glycerol utilisation but not the major contribution to glycerol uptake into the cell, possibly reflecting an impact on transport into glycosomes, where glycerol is utilised [25].

### *T*. *b*. *brucei aqp*-null cells display a glycerol efflux defect and respiratory inhibitor-sensitivity

**Fig 4:**
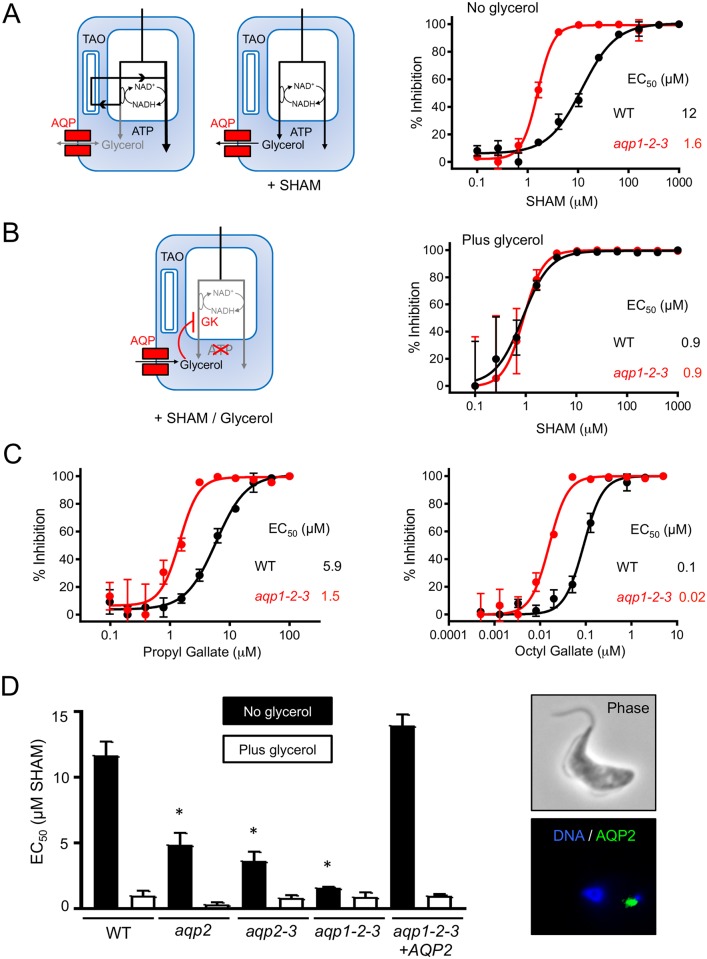
*aqp*-null *T*. *b*. *brucei* display defective glycerol-efflux and respiratory inhibitor-sensitivity. (A) Bloodstream *T*. *brucei* express a SHAM-sensitive mitochondrial trypanosome alternative oxidase (TAO). Under aerobic conditions, TAO activity allows ATP production without glycerol production as indicated by the black lines (left-hand blue ‘cell’). SHAM blocks TAO-activity, leading to the anaerobic production of glycerol, which is toxic if not removed, as indicated by the black lines (right-hand blue ‘cell’). SHAM dose-response curves for wild-type (WT) and *aqp1-2-3* null-cells. EC_50_ values are indicated. (B) In the presence of SHAM and glycerol, the glycerol inhibits glycerol kinase (GK), also preventing ATP-production by the anaerobic route (blue ‘cell’). SHAM dose-response curves as in A but in the presence of 10 mM glycerol. (C) Propyl gallate and octyl gallate dose-response curves for wild-type (WT) and *aqp1-2-3* null-cells. EC_50_ values are indicated. (D) SHAM EC_50_ values +/- 10 mM glycerol from A-B and also from *aqp2*, *aqp2-3* and *aqp1-2-3* cells re-expressing ^GFP^AQP2. * indicates significantly different (*P*<0.01) to WT using an ANOVA test in GraphPad Prism. Pairwise comparisons +/- glycerol, except in the case of the *aqp1-2-3* null, indicated significant (*P* <0.001) differences using a Student’s *t*-test. Error bars, SD. The images to the right show re-expression of ^GFP^AQP2 in *aqp1-2-3* null-cells.

We next asked whether *aqp*-defective trypanosomes displayed glycerol-efflux defects as well as the glycerol-uptake defects described above. Salicylhydroxamic acid (SHAM) increases intracellular glycerol levels by inhibiting the trypanosome alternative oxidase (TAO) [26], a ubiquinol oxygen oxidoreductase that is cyanide-insensitive and maintains redox balance as part of the glycerol-3-phosphate oxidase system (see Fig 4A, left-hand panels). Consistent with a glycerol-efflux defect, dose-response curves revealed that *aqp1-2-3* null-cells were SHAM-sensitive (EC_50_ decreased >7-fold) relative to wild-type cells (Fig 4A, right-hand panel: EC_50_ 1.6 and 12 μM, respectively). SHAM plus glycerol rapidly kills bloodstream-form African trypanosomes [27] (see Fig 4B, left-hand panel), but we predicted that the impact of added glycerol would not be pronounced in glycerol-uptake defective *aqp1-2-3* null-cells. Indeed, SHAM dose-response curves generated in the presence of 10 mM glycerol (Fig 4B, right-hand panel) revealed a substantial impact of glycerol against wild-type cells but only a very weak impact against *aqp1-2-3* null-cells; glycerol reduced SHAM EC_50_ values by 13 and 1.8-fold, respectively; to 0.9 μM in both cases (compare Fig 4A and 4B). We also tested the additional TAO inhibitors, propyl gallate and octyl gallate [28], against wild-type and *aqp1-2-3* null-cells. Once again, and consistent with a glycerol-efflux defect, dose-response curves revealed that *aqp1-2-3* null-cells were TAO inhibitor sensitive relative to wild-type cells (Fig 4C); EC_50_ was reduced by 4-fold and 5-fold, respectively.

Since our glycerol-utilisation assays indicated a defect in *aqp2* null *T*. *b*. *brucei*, we next asked whether these cells also displayed increased sensitivity to SHAM, consistent with a glycerol-efflux defect. We also tested SHAM-sensitivity in *aqp2-3* null cells and in *aqp1-2-3* null cells re-expressing *AQP2*; re-expressed AQP2 was localised to the flagellar pocket (Fig 4D, right-hand side), as expected [9]. The full set of SHAM (plus glycerol) EC_50_ values are shown in Fig 4D. SHAM-sensitivity was indeed observed in *aqp2* null (2.4-fold) *aqp2-3* null (3.2-fold) and *aqp1-2-3* null cells (see above); these cells were all significantly more sensitive to SHAM than wild type (Fig 4D), and AQP2 re-expression effectively reversed SHAM-sensitivity in the *aqp1-2-3* null background (Fig 4D). Also, 10 mM glycerol reduced SHAM EC_50_ values to <1 μM in all cell types and this reduction was significant in all but the *aqp1-2-3* null cells (Fig 4D), again consistent with almost complete ablation of glycerol transport in the latter case only.

### Melarsoprol-resistant clinical isolates display respiratory inhibitor-sensitivity

TAO inhibitor-sensitivity in *aqp*-null *T*. *b*. *brucei* may help to predict how trypanosomes in patients will respond to respiratory inhibitors. In particular, naturally occurring melarsoprol-resistant clinical *T*. *b*. *gambiense* isolates display chimerisation of the *AQP2/3* genes [14]. Indeed, a substantial proportion, >50% in some areas, of circulating *T*. *b*. *gambiense* may be *AQP2*-defective [12,13]; probably due to selection with melarsoprol since the 1940s. To analyse whether this *AQP2* defect might have an impact on respiratory inhibitor-sensitivity in clinical isolates, we generated SHAM dose-response curves. The isolates selected were the melarsoprol/pentamidine sensitive STIB930 and STIB891 strains (EC_50_ <10 and <2 nM, respectively, according to [12]), the melarsoprol/pentamidine resistant K03048 and 40 AT isolates from melarsoprol-relapsed patients (EC_50_ >20 and >50 nM, respectively, according to [12]) and a 40 AT-derivative that re-expresses *AQP2* and is consequently restored to melarsoprol/pentamidine sensitivity [14]. The STIB930 and STIB891 strains are from patients in Côte d'Ivoire in 1978 [29] and Uganda in 1995 [30] and the K03048 and 40 AT isolates are from patients in South Sudan in 2003 [31] and the Democratic Republic of the Congo in 2006 [13], respectively. The STIB930 and STIB891 strains have intact *AQP2* genes, while neither of the latter isolates has an intact *AQP2* gene [12].

**Fig 5:**
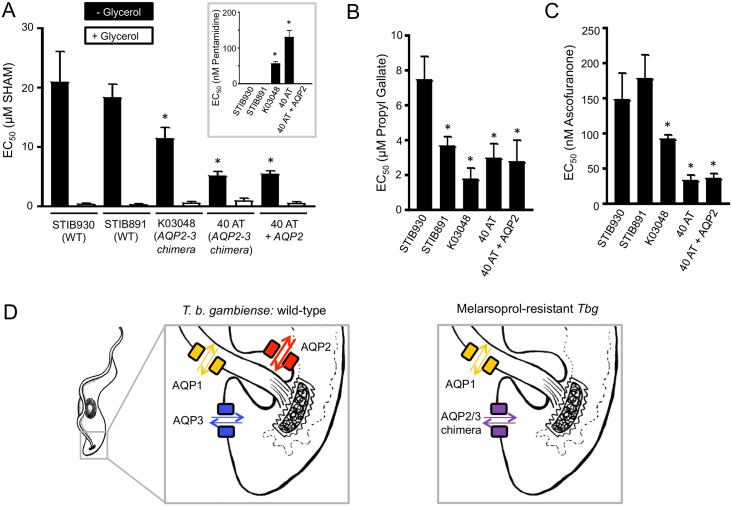
Respiratory inhibitor-sensitivity in *T*. *b*. *gambiense* isolates and AQP-mediated glycerol transport. (A) SHAM EC_50_ values for the *T*. *b gambiense* strains are indicated +/- glycerol. The inset shows pentamidine EC_50_ values. * indicates significantly different (*P*<0.05) to STIB930 using an ANOVA test in GraphPad Prism. All pairwise comparisons +/- 10 mM glycerol also indicated significant (*P* <0.001) differences using a Student’s *t*-test. Error bars, SD. (B) Propyl gallate and (C) Ascofuranone EC_50_ values. Other details as in A. (D) Model for glycerol transport by AQPs in *T*. *b*. *gambiense*. The weight of the arrows indicates relative impact on glycerol utilisation and efflux, with AQP2 being the major contributor; note that transport across both the plasma and glycosomal membranes contributes to glycerol utilisation and efflux, see the text for more details. The right-hand panel indicates the situation in melarsoprol-resistant (reduced melarsoprol uptake) and SHAM-sensitive (reduced glycerol efflux) clinical isolates where a chimeric AQP2/3 replaces AQP2 and AQP3.

As our studies on *T*. *b*. *brucei* had predicted, dose-response curves for the *T*. *b*. *gambiense* strains revealed significantly lower EC_50_ values for both *aqp2*-defective strains relative to the *AQP2* controls (Fig 5A); the strains that lacked AQP2 were also confirmed to be pentamidine-resistant (Fig 5A, inset), as previously reported [12]. These results suggest a glycerol-efflux defect in the *aqp2*-defective clinical isolates. Re-expression of *AQP2* in 40 AT cells did not significantly alter SHAM-sensitivity, however (Fig 5A). This may indicate that the AQP2/3 chimera interferes with glycerol efflux by recombinant AQP2, possibly due to the formation of AQP hetero-tetramers that, despite the glycerol efflux defect, continue to contribute to pentamidine uptake by endocytosis [22]. The addition of 10 mM glycerol significantly reduced SHAM EC_50_ values to <1 μM in all five cell-types (Fig 5A), indicating, as predicted, continued glycerol influx in each case. To extend these findings, we examined the impact of two additional TAO inhibitors, propyl gallate and ascofuranone [32], on the same set of strains described above. Dose-responses for propyl gallate (Fig 5B) and ascofuranone (Fig 5C) revealed similar EC_50_ profiles as detailed above for SHAM. Although the STIB891 EC_50_ for propyl gallate was relatively low, both *aqp2*-defective strains displayed an even lower EC_50_, and both were significantly more sensitive to the respiratory inhibitors than the STIB930 control (Fig 5B and 5C). Once again, re-expression of *AQP2* in 40 AT cells did not significantly alter respiratory inhibitor sensitivity (Fig 5B and 5C).

Together, our results indicate that triple *aqp*-null and *aqp2* null *T*. *b*. *brucei* exhibit defects in bidirectional glycerol flux. The evidence is three-fold; first, failure to take up or effectively utilise glycerol as a carbon source; second, sensitivity to multiple respiratory inhibitors which produce toxic levels of intracellular glycerol; and third, no significant increase in SHAM-sensitivity in excess glycerol in triple-null cells. Thus, glycerol flux appears to be almost absent in *aqp1-2-3* triple-null cells. Our results also indicate that AQP2 makes a key contribution to glycerol utilisation and efflux. This interpretation is supported by a substantial defect in glycerol utilisation and sensitivity to SHAM in *aqp2* null-cells; a phenotype that is reversed by AQP2 re-expression in *aqp1-2-3* triple-null cells. Importantly, analysis of melarsoprol/pentamidine sensitive *T*. *b*. *gambiense* reference strains and melarsoprol/pentamidine resistant clinical-isolates supports the idea that AQP2 also makes a key contribution to glycerol efflux in trypanosomes in patients (see Fig 5D). We propose that it is the replacement of *AQP2* with the *AQP2-3* chimera in clinical isolates (Fig 5D) that increases sensitivity to respiratory inhibitors. Notably, although the chimera comprises <15% of the AQP3-sequence at the *C*-terminus, like AQP3 [9], the chimera is distributed within the plasma membrane [10]; AQP2 by contrast is concentrated in the flagellar pocket in bloodstream-form cells [9].

## Discussion

Here, we describe bloodstream-form *T*. *b*. *brucei* strains that lack all three AQPs. These strains exhibit only a minimal fitness-defect and no apparent osmoregulation-defect. They do, however, exhibit bidirectional defects in glycerol transport. AQP2 is an important determinant of cross-resistance to melarsoprol and pentamidine and this AQP was also found to make a key contribution to glycerol transport. Finally, following analysis of clinical isolates, we propose that the AQPs behave similarly in parasites in patients, suggesting that TAO-inhibitors may be more effective against melarsoprol-resistant African trypanosome infections.

The triple *aqp*-null strain was assembled with the primary purpose of dissecting AQP-functions. We note though that successful generation of such a strain indicates that the AQPs are unlikely to be suitable therapeutic targets for inhibition. It was also possible to generate malaria parasites that lacked the single encoded *AQP* gene; these *aqp*-null *Plasmodium* parasites displayed defective glycerol uptake and moderately reduced virulence [33]. We find that *aqp1-2-3* null *T*. *b*. *brucei* establish parasitemia in mice. Indeed, strains isolated from patients following melarsoprol treatment-failure, in an area where treatment-failure is common, display fusion of *AQP2* and *AQP3* to form an *AQP2/3* chimera [12,13]. This suggests, either that these *AQPs* are dispensable at all stages of the life-cycle, or that the chimera complements the defect(s). It remains possible that AQP1 or the AQP2/3 chimera have essential functions in other life-cycle stages, but we were able to differentiate triple-null cells to the procyclic stage *in vitro* and also note that *T*. *vivax* and *T*. *congolense* appear to lack both the *AQP1* and *AQP2* genes [34].

The three *T*. *b*. *brucei* AQPs were previously reported to play a role in osmoregulation [16]. The same study indicated an additional glycerol transport activity in *T*. *b*. *brucei* [16]. In contrast, we observe minimal or no defect in osmoregulation and detected minimal residual glycerol flux in triple *aqp*-null cells. The former difference could potentially reflect adaptation in null cells but the latter difference is likely explained by only 36% AQP2 knockdown or 73% triple AQP knockdown in the former study [16]. Notably, adaptation, if it operates, would also be expected in clinical and veterinary isolates that lack *AQP* genes. How is osmoregulation achieved in other parasitic trypanosomatids? A contractile vacuole/spongiome complex is present in *Trypanosoma cruzi* and *Leishmania major*, and aqua(glycero)porins have been localised to these organelles [35,36]; the *T*. *cruzi* aquaporin is not closely related to the *T*. *brucei* AQPs but *Leishmania* AQP1 is closely related [6] and does play a role is osmoregulation [37]. However, water can diffuse across membranes and alternative mechanisms of osmoregulation do operate. In both *L*. *major* [38] and *Crithidia luciliae* [39], cells tolerate hypotonic stress through the efflux of amino acids and, in *Leishmania donovani*, also through the efflux of inorganic osmolytes [40]. Thus, *T*. *brucei* AQPs may contribute to osmoregulation, but we suggest that the primary roles of these AQPs in bloodstream-form cells are the transport of glycerol and other solutes.

Under aerobic conditions, *T*. *b*. *brucei* can use glycerol as a carbon source [41]. We found that triple *aqp*-null cells, and even *aqp2*-null cells, fail to effectively utilise glycerol. This indicates that AQPs contribute to glycerol-uptake and utilisation and that AQP2 makes a key contribution. Since glycerol utilisation and production under anaerobic conditions occurs inside glycosomes [25], we must consider glycosomal transport as well as transport across the plasma membrane. A *T*. *cruzi* aquaporin is localised to acidocalcisomes [36] but AQPs have not been reported to be associated with glycosomes. It is possible that the *T*. *brucei* AQPs are also present in glycosomal membranes but there may equally be alternative glycerol transporters associated with these organelles.

Carbohydrate catabolism in African trypanosomes has been considered a promising potential antitrypanosomal therapeutic target for >40 years. Indeed, a SHAM plus glycerol combination blocks aerobic and anaerobic glycolysis *in vivo* and clears parasites from the blood of experimental animals within 5 min [27]. Since this combination is so effective, glycerol-efflux has remained of particular interest [26]. SHAM inhibits TAO, which is upregulated in the bloodstream-form and not found in other trypanosomatids or in the mammalian host [26]. TAO inhibition blocks the aerobic pathway and increases the production of ATP via the reverse-action of glycerol kinase [41]. The glycerol produced by this anaerobic glycolysis will become toxic if not removed from the cell. If glycerol is not removed, it reverses the action of glycerol kinase by mass-action and also blocks the anaerobic pathway, explaining the toxic effect of SHAM plus glycerol. Our findings indicate that this SHAM-glycerol effect is dependent upon the AQPs. Indeed, our results show that *aqp2*, *aqp2-3* and *aqp1-2-3* cells, and clinical isolates lacking *AQP2* but with an *AQP2/3* chimera, display increased sensitivity to multiple respiratory inhibitors in the absence of exogenous glycerol. Thus, AQP2 plays a key role in both glycerol utilisation and efflux.

The combination of SHAM with a large dose of glycerol, required at up to 15 g per kg, remains impractical as a therapy [42]. More potent antitrypanosomal TAO inhibitors have been developed, however [42,43,44]. Our finding, therefore, that *aqp2*-deficiency is associated with TAO-inhibitor sensitivity, has implications for potential future therapeutic strategies. For example, new TAO-inhibitors may be effective as mono-therapies against melarsoprol-resistant *T*. *b*. *rhodesiens*e [4], or *T*. *b*. *gambiens*e, known to lack *AQP2* in the latter case [12]. This may also be the case for *T*. *vivax* and *T*. *congolense*, where the reference genomes indicate the absence of both the *AQP1* and *AQP2* genes and the presence of only an *AQP3*-like gene (Tvy486_1013610 and TcIL3000_10_12040, respectively) [34]. Indeed, although SHAM alone is ineffective against *T*. *vivax* [45], ascofuranone is effective against *T*. *vivax* infections in mice without added glycerol [32]. This and other TAO inhibitors are thought to function by mimicking ubiquinol and blocking electron transfer to the oxidase [46].

Melarsoprol has been highly effective against trypanosomiasis but clinical resistance, due to an *aqp2*-defect, has become widespread [12]. An option, therefore, could be to apply TAO-inhibitors and melarsoprol sequentially or in combination; this could establish a counter-resistance approach whereby AQP2 is required for both the uptake and efflux of toxins. Further similar options may emerge from on-going efforts to develop safer and orally available arsenical formulations [47]. Ultimately, reciprocal shifts in drug-sensitivity, such as the example we describe here, may be exploited to develop novel paradigms of targeted-therapy. Such strategies could restrict or even reverse the emergence and spread of drug resistance in human and livestock parasites, which would be of great value given the high cost of developing new therapies.

Our studies on *aqp*-null *T*. *b*. *brucei* and on clinical isolates of *T*. *b*. *gambiense* have revealed bidirectional defects in glycerol transport and the key contribution of AQP2, the AQP specifically responsible for melarsoprol- and pentamidine-sensitivity, now also shown to impact respiratory inhibitor sensitivity. Thus, AQPs impact the efficacy of three major classes of antitrypanosomal drugs. These new mechanistic insights into differential sensitivities to antitrypanosomal drugs, in both clinical and veterinary settings, are potentially exploitable.

## Materials and methods

### *T*. *b*. *brucei* growth and manipulation

Bloodstream-form *T*. *brucei*, Lister 427, MiTat 1.2, clone 221a, and all derivatives were cultured in HMI-11 as previously described [48]. Bloodstream-form *T*. *b*. *gambiense* were cultured in the same media but with 15% FCS and 5% human serum. 2T1 [24], *aqp2* [9], *aqp2-3* [23], STIB930, STIB891, K03048, 40 AT [12] and 40 AT plus AQP2 [14] strains were described previously. SHAM, glycerol, octyl gallate and propyl gallate were from Sigma. SHAM was dissolved in DMSO, the gallates were dissolved in 70% ethanol or DMSO and ascofuranone was dissolved in DMSO. EC_50_ assays were performed using the AlamarBlue method as described [49] with 10 mM glycerol added as appropriate; drug exposure was for 66–67 h and AlamarBlue incubation was for 5–6 h. Plates were read on an Infinite 200 Pro plate-reader (Tecan). Growth rates in culture were monitored by splitting to 1 x 10^5^ cells/ml and by counting daily. Three Balb/c mice were infected with *aqp1-2-3* triple-null trypanosomes by intraperitoneal injection of 10^4^ cells in 0.2 ml of growth medium. Parasitaemia was determined daily following tail bleeds. Mice were purchased from Envigo, UK. Differentiation to insect-stage, procyclic form cells was initiated by washing 2 x 10^7^ cells twice in DTM [50] and re-suspending in 5 ml DTM supplemented with citrate (3 mM) and cis-aconitate (3 mM) at 27°C.

### Plasmids and strain construction

For *AQP*-knockout plasmid constructs, AQP-flanking sequences were inserted on both sides of selectable marker cassettes. Restriction enzyme cleavage at the distal ends of the AQP targeting regions was used to linearise plasmid constructs prior to transfection. The AQP2-3 locus was disrupted by replacing a 4,772 bp fragment [9] with *BLA* and a modified *NPT* selectable marker cassette. The *AQP1* locus was disrupted by replacing a 647 bp fragment with *NPT* and (a modified) *PAC* selectable marker cassettes. The *AQP1*:*PAC* and *AQP2-3*:*NPT* cassettes were modified using annealed oligonucleotides (XSceF: CTAGTAGGGATAACAGGGTAAT, and XSceR: CTAGGATTACCCTGTTATCCCTA) to engineer an I-*Sce*I site at an *Xba*l site adjacent to each 5’-targeting region. Other oligonucleotide sequences are available upon request.

During creation of the *aqp1-2-3* triple-null strains, selectable markers were recovered using I-*Sce*I meganuclease-induction in a 2T1 (*BLE*:*PAC*) background [48]. Briefly, a pRPa^Sce^ [51] construct (*HYG* recovers *PAC*) was introduced at the tagged locus on chromosome 2 and the *AQP2-3* alleles were replaced with *BLA* and *NPT*-cassettes, the latter containing the flanking I-*Sce*I cleavage site. Induction with 1 μg.ml^-1^ tetracycline triggered I-*Sce*I cleavage and duplication of the *BLA*-cassette (*NPT* recovered). A similar process was repeated for *AQP1* alleles but this time with *NPT* and *PAC*-cassettes (*PAC* recovered). The ph3E construct [48] was then used to remove the I-*Sce*I cassette (*PAC* recovered *HYG*). A pRPa^AQP2^ construct (*HYG* recovers *PAC*) was then used for expression of recombinant *AQP2* in the 2T1-*aqp1-2-3* null-background (*BLE*:*BLA*:*NEO*:*PAC*). Selectable-marker recovery was confirmed by screening individual clones in multi-well plates. Strains were transfected using a Nucleofector (Lonza) and cytomix. Transformants were selected with phleomycin (1 μg.ml^-1^), blasticidin (10 μg.ml^-1^), G418 (2 μg.ml^-1^), puromycin (2 μg.ml^-1^) and hygromycin (2.5 μg.ml^-1^) as appropriate and *AQP* knockout was confirmed by Southern blotting, carried out according to standard protocols.

### Hypo-osmotic shock assays

Cell volume during hypo-osmotic shock was assessed using a light-scattering assay. Briefly, 5 x 10^7^ cells were pelleted and resuspended in ice cold Earle’s salt buffer (116 mM NaCl, 1.8 mM CaCl_2_, 5mM KCl, 0.8 mM MgSO_4_, 1 mM NaH_2_PO_4_, 30 mM HEPES, 30 mM glucose, pH 7.4). 1.3 x10^4^ cells in 100 μl per well were added to 96-well plates. Either 100 μl of cold deionised water (hypo-osmotic) or Earle’s salt buffer (iso-osmotic) was added to each well. Results were then immediately read at 18-s intervals over a course of 25-min, using a Tecan Infinite 200 pro plate-reader at 595 nM absorbance.

### Microscopy

For phase and fluorescence microscopy, cells were fixed in 1% paraformaldehyde, settled onto slides and mounted in Vectashield (Vector Laboratories) containing 4,6-diamidino-2-phenylindole (DAPI). Images were captured using an Axiovert 200 epifluorescence microscope in conjunction with an Axiocam 105 colour camera (Zeiss) and were processed using Zen digital imaging suite.

### ATP quantification

We used the CellTiter-Glo luminescence assay (Promega). Briefly 5 x 10^6^ cells were washed twice with cold PBS and re-suspended in 1 ml of 37°C PBS with either 5 mM glucose or glycerol in PBS for 20-min before performing the assay as per the manufacturers’ instructions. Plates were read on an Infinite 200 Pro plate-reader (Tecan). Values were compared to an ATP standard-curve.

### Glycerol uptake assay

We used a [^14^C] glycerol centrifugation method [52] with minor modifications. Briefly, cells were pelleted by centrifugation (1,000 g, 10 min), washed twice in transport buffer (33 mM HEPES, 98 mM NaCl, 4.6 mM KCl, 0.55 CaCl_2_, 0.07 MgSO_4_, 5.8 mM Na_2_PO_4_, 0.3 mM NaHCO_3_, 14 mM glucose, pH 7.3) and diluted to 1 x 10^8^/ml in transport buffer on ice. Uptake was measured (at 37°C) by introducing 100 μl of cells to 100 μl transport buffer, containing 0.25 uCi glycerol. This reaction mixture was immediately loaded onto 100 μl of dibutyl phthalate (Sigma) in 1.5 ml Eppendorf tubes. After incubation for 5 min, cells were pelleted through the oil layer by centrifugation (16,000g, 1 min). The tubes were then frozen on liquid nitrogen and the bottoms of the tubes, containing pellets, were snipped directly into scintillation vials. Pellets were solubilised overnight in 150 μl 1 M NaOH, before mixing with 2 ml of scintillation fluid and reading on a scintillation counter (Beckman LS 6500) for 1 min.

### Ethics statement

All animal experiments were approved by the Ethical Review Committee at the University of Dundee and performed under the Animals (Scientific Procedures) Act 1986 (UK Home Office Project Licence PPL 70/8274) in accordance with the European Communities Council Directive (86/609/EEC).
